# Structural understanding of the recycling of oxidized ascorbate by dehydroascorbate reductase (OsDHAR) from *Oryza sativa* L. *japonica*

**DOI:** 10.1038/srep19498

**Published:** 2016-01-18

**Authors:** Hackwon Do, Il-Sup Kim, Byoung Wook Jeon, Chang Woo Lee, Ae Kyung Park, Ah Ram Wi, Seung Chul Shin, Hyun Park, Young-Saeng Kim, Ho-Sung Yoon, Han-Woo Kim, Jun Hyuck Lee

**Affiliations:** 1 Division of Polar Life Sciences, Korea Polar Research Institute, Incheon 406-840, Republic of Korea; 2 School of Life Sciences, BK21 Plus KNU Creative BioResearch Group, Kyungpook National University, Daegu 702-701, Republic of Korea; 3 Department of Polar Sciences, University of Science and Technology, Incheon 406-840, Republic of Korea; 4 Division of Biological Sciences, University of California San Diego, La Jolla, California 92093-0116, USA

## Abstract

Dehydroascorbate reductase (DHAR) is a key enzyme involved in the recycling of ascorbate, which catalyses the glutathione (GSH)-dependent reduction of oxidized ascorbate (dehydroascorbate, DHA). As a result, DHAR regenerates a pool of reduced ascorbate and detoxifies reactive oxygen species (ROS). In previous experiments involving transgenic rice, we observed that overexpression of *DHAR* enhanced grain yield and biomass. Since the structure of DHAR is not available, the enzymatic mechanism is not well-understood and remains poorly characterized. To elucidate the molecular basis of DHAR catalysis, we determined the crystal structures of DHAR from *Oryza sativa* L. *japonica* (OsDHAR) in the native, ascorbate-bound, and GSH-bound forms and refined their resolutions to 1.9, 1.7, and 1.7 Å, respectively. These complex structures provide the first information regarding the location of the ascorbate and GSH binding sites and their interacting residues. The location of the ascorbate-binding site overlaps with the GSH-binding site, suggesting a ping-pong kinetic mechanism for electron transfer at the common Cys20 active site. Our structural information and mutagenesis data provide useful insights into the reaction mechanism of OsDHAR against ROS-induced oxidative stress in rice.

Ascorbate (ascorbic acid or vitamin C; AsA) is a well-known cofactor in many enzymatic reactions and is important for defence mechanisms against oxidative stress. However, AsA is continuously oxidized by the scavenging reactive oxygen species (ROS) generated by environmental stressors such as drought, salinity, ultraviolet (UV) light, and extreme temperatures. It has been reported that the overexpression of *DHAR* increased grain yield and biomass in transgenic sweet potato, rice, tomato, acerola, and tobacco plants^1^,^2^,^3^,^4^,^5^,^6^,^7^.

As a major antioxidant, AsA can directly scavenge free radicals and is present in two different oxidized forms, dehydroascorbate (DHA) and monodehydroascorbate (MDHA). The regeneration system for AsA is very important for maintaining AsA homeostasis against exogenous stimuli in living organisms. In plants, there are two key enzymes involved in AsA recycling known as dehydroascorbate reductase (DHAR) and monodehydroascorbate reductase (MDHAR). MDHA is the primary oxidation product and is the one-electron oxidized form of AsA^8^. Two MDHA molecules can react with each other to form one molecule each of AsA or DHA, a two-electron oxidized form of AsA. DHAR catalyses the reduction of DHA to AsA using glutathione (GSH) as a hydrogen donor before the spontaneous hydrolysis of DHA to irreversibly form 2,3-diketogulonic acid. In contrast, MDHAR utilizes NAD(P)H as an electron donor to reduce MDHA before spontaneous oxidation of MDHA to form DHA^9^ (Fig. 1).

To date, the DHAR structure has not been deposited in the RCSB Protein Data Bank (PDB). However, DHAR shares significant sequence similarity with chloride intracellular channels (CLICs) (~26% identity and ~46% similarity) and the omega-class glutathione S-transferase (GSTO) (~21% identity and 35% similarity); furthermore, CLIC1 and GSTO2 have DHAR activity^10^,^11^,^12^,^13^. In addition, DHAR, CLIC1, and GSTOs commonly contain a highly conserved catalytic cysteine residue and GSH binding residues near the active site. Interestingly, CLIC1 exists in both soluble (monomeric state) and integral membrane (non-covalent dimeric state) forms; soluble CLIC1 undergoes a transition to a membrane-bound form through oligomerization followed by the formation of an intramolecular disulfide bond (Cys24-Cys59)^12^,^14^. In humans, only GSTOs (GSTO1-1 and GSTO2-2) have DHAR activity. Multiple sequence alignment analyses revealed that GSTO1-1 and GSTO2-2 contain a conserved catalytic cysteine residue and GSH binding sites, while other classes of GSTs have different amino acids in these positions, explaining why only GSTOs show DHAR activity^10^,^13^.

We previously reported that transgenic rice expressing the *DHAR* gene from *Oryza sativa* L. *japonica* (*OsDHAR*) showed increased amounts of AsA and higher crop productivity than wild-type (WT) rice^6^. These results strongly suggest that AsA pools are closely associated with grain yield and decreased ROS in plants can affect and improve environmental adaptation and production. To expand our knowledge of the underlying molecular mechanism of OsDHAR activity against ROS, high-resolution crystal structures of the apo-form, AsA-bound, and GSH-bound OsDHAR were examined at 1.9, 1.7, and 1.7 Å resolution, respectively. In addition, we performed site-directed mutagenesis and biochemical analysis based on the structural information, which revealed that the Cys20 and Lys8 residues are important for enzymatic activity. Structural comparison studies revealed that Cys20 is a catalytic residue and that Lys8 directly interacts with AsA and GSH to reduce Cys20. In conclusion, we propose an enzymatic reaction scheme of OsDHAR for AsA regeneration against ROS.

## Result and Discussion

### Overall structure of apo and ascorbate-bound OsDHAR

**Figure 2:**
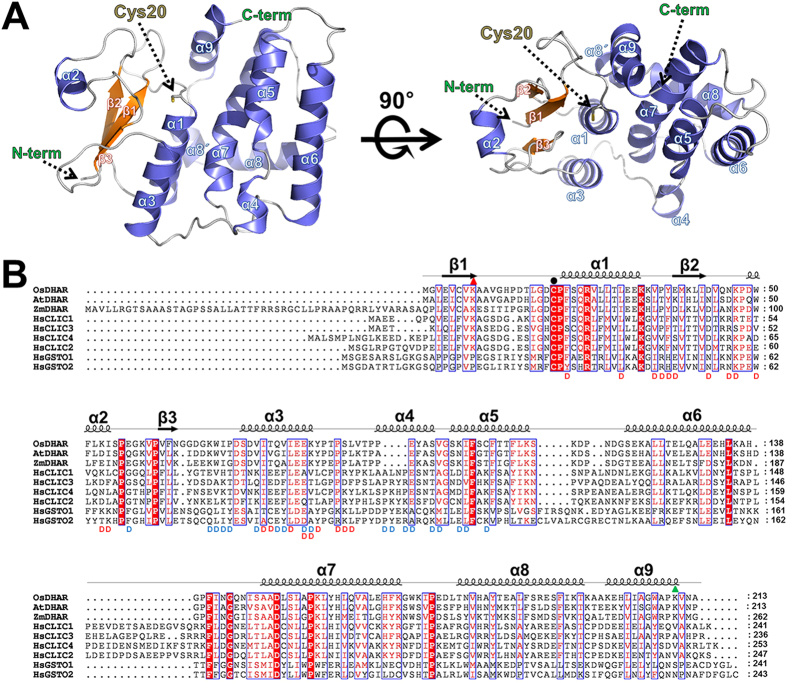
Crystal structure of OsDHAR and multiple sequence alignment with CLICs and GSTOs. **(A)** Ribbon diagram showing the structure of the reduced apo form of OsDHAR. The β-strands are shown as orange arrows, α-helices as slate blue, and connecting loops as gray coils. The catalytic active site residue, Cys20, is labelled and shown as a stick model. The N- and C-termini are labelled. **(B)** Multiple sequence alignment of OsDHAR, CLICs, and GSTOs. Aligned sequences include OsDHAR (UniProtKB code Q65XA0), AtDHAR (UniProtKB code Q9FWR4), ZmDHAR (UniProtKB code B6U098), human CLIC1 (UniProtKB code O00299), human CLIC2 (UniProtKB code O15247), human CLIC3 (UniProtKB code O95833), human CLIC4 (UniProtKB code Q9Y696), human GSTO1 (UniProtKB code P78417), and human GSTO2 (UniProtKB code Q9H4Y5). Alignment positions identical in all sequences are marked with a red background. The secondary structures obtained from the crystal structure are shown above the aligned sequences. The Lys8 and Lys210 residues of OsDHAR are indicated above the alignment residues with red and green triangles, respectively. Residues participating in dimer interactions of hGSTO1-1 (PDB code 4IS0) and hCLIC1 (PDB code 1RK4) are indicated by blue “D” and red “D”, respectively.

The crystal structure of the native form of OsDHAR (UniProtKB code Q65XA0; 213 amino acid residues) was determined by molecular replacement at 1.9 Å resolution. Most of the residues were well-resolved, except for the N-terminal methionine residue. The structure shows that the asymmetric unit contains one molecule of OsDHAR and adopted a canonical GST fold, consisting of three β-strands and three α-helices in the N-terminal subdomain (residues 4–84) and six α-helices in the C-terminal subdomain (residues 94–210), although OsDHAR showed modest sequence identity (~20%) with GSTs (Fig. 2). The closest structural relative was found to be human CLIC1 (PDB code 1K0O), in which 156 C_α_ could be aligned with a root-mean-square deviation (RMSD) value of 0.93 Å, followed by GSTO1 from *Homo sapiens* (PDB code 3LFL) (122 aligned C_α_ with an RMSD of 2.07 Å) and the putative stringent starvation protein A from *Burkholderia cenocepacia* (PDB code 4QQ7) (122 aligned Cα with an RMSD of 2.14 Å)^12^,^15^. Notably, OsDHAR was found to be a monomer under both reducing and oxidizing (10 mM H_2_O_2_) conditions (Supplemental Fig. S1 online). In contrast, GSTs showed a preference for dimer formation, and CLICs formed dimers in their oxidized states^16^. CLIC1 undergoes a large conformation change upon oxidation. This structural change is reversible and involves the formation of an intramolecular disulfide bond and a non-covalent dimer. The dimer structure of CLIC1 has a large hydrophobic surface, which can be used for membrane incorporation and chloride ion channel formation^15^,^16^,^17^. Littler *et al*. proposed that the N-terminal region (residues 24–46 of CLIC1) may form a transmembrane helix in the chloride ion channel^17^. In the case of GSTs, the enzymes are dimeric, with each monomer equipped with a catalytic domain. Several classes of GST have been shown to exhibit non-catalytic ligand binding in the dimer interface^13^,^15^. Recently, Brock *et al*. showed that the non-catalytic ligand binding site (ligandin site) is in the dimeric cleft of hGSTO1-1 created by largely hydrophobic residues^18^. These results indicate that the dimer formation of GSTOs and CLICs is very important for their biological function. Sequence alignment results showed that the dimerization sites were not conserved between omega GSTs and CLICs (Fig. 2), explaining why these two proteins have completely different dimeric structures (Supplemental Fig. S2 online).

**Figure 3:**
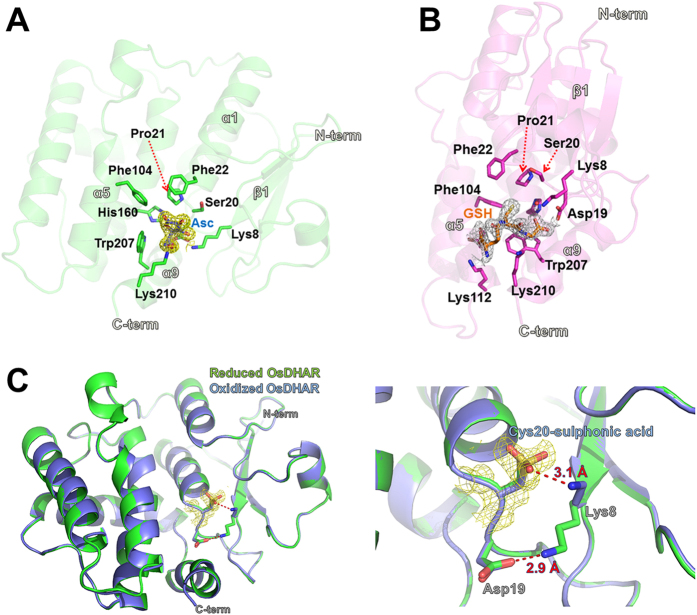
Substrate binding and active sites of OsDHAR. **(A)** Ribbon diagram representing the AsA-binding site of OsDHAR. Bound AsA is depicted in slate blue with a 2Fo-Fc electron density map contoured around it at the 1.0 σ level. **(B)** Ribbon diagram representing the GSH binding site of OsDHAR. Bound GSH is depicted in orange with a 2Fo-Fc electron density map contoured around it at the 1.0 σ level. **(C)** Structural comparison between reduced and oxidized (slate blue) OsDHAR. Right panel shows a magnified view of the superimposed active site of reduced and oxidized OsDHAR. A 2Fo-Fc map is shown around the Cys20-sulfonic acid contoured at the 1.0 σ level. A conformational change of Lys8 occurred, and a new hydrogen bond was formed in the oxidized OsDHAR between the NZ group of Lys8 and the side-chain oxygen of Cys20-sulfonic acid.

**Figure 4:**
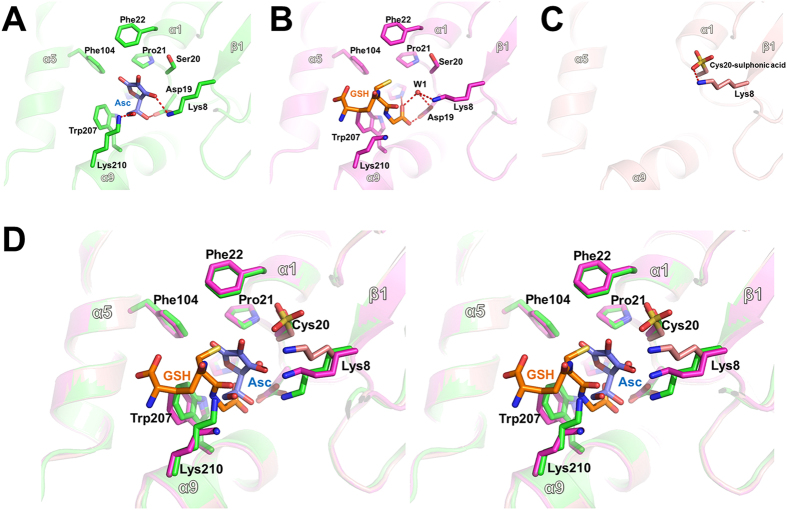
Structural comparison around the active site residues of AsA-bound, GSH-bound, and oxidized OsDHAR. **(A)** Close-up view showing the AsA-binding site. Residues involved in AsA binding are shown as stick models using the same colour code as shown in Fig. 3A. The dashed red lines indicate hydrogen bonds. **(B)** Close-up view showing the GSH-binding site. Residues involved in GSH binding are shown as stick models using the same colour code as shown in Fig. 3B. (**C**) Close-up view around the oxidized Cys20 residue. The Lys8 residue interacts with the sulfonic acid of Cys20. **(D)** Stereodiagram of superimposed OsDHAR structures with AsA, GSH, and oxidized Cys20 residue. The figure orientation here is similar to those in Fig. 4A–C. The conformational changes of Lys8 and Lys210 are clearly shown by structural superposition.

After inspection of the apo-OsDHAR structure, we predicted that the highly conserved Cys20 residue acts as the catalytic nucleophile and found that this residue is located at the cleft between the N- and C-terminal subdomains. To obtain crystals of the AsA-bound protein, Cys20-mutated OsDHAR (C20S) was used, which lacks reductase activity but exhibits AsA binding activity. The finding was similar to our results for the DHAR mutant (C20S) in *Populus tomentosa* DHAR2 (PtDHAR2). Alanine replacement of Cys20, which was predicted to be the GSH binding site in the PtDHAR2 protein, eliminated DHAR activity^19^. The AsA-bound OsDHAR structure was refined at 1.7 Å resolution and contained one molecule of AsA bound to OsDHAR in the asymmetric unit (Fig. 3A). The AsA bound to a pocket at the cleft that separated the N- and C-domains of OsDHAR. In detail, the 3′-OH and 6′-OH groups on AsA formed hydrogen bonds with the side chain of the Lys8 and Lys210 residues, respectively. In addition, the 5′-OH group of AsA interacted with the OD2 atom of Asp19. The C1′-carbon of the bound AsA was 5.0 Å from Ser20, but rotation of the side chain may produce a suitable distance for catalytic reduction by the Cys20 residue (Fig. 4A). Structural analyses also revealed that OsDHAR undergoes AsA-associated conformational changes. In particular, the Lys8, Trp207, and Lys210 residues showed structural differences from their corresponding residues compared with the oxidized and GSH-bound structures, and rearrangement of these residues is related to the movements necessary for the interactions involved in AsA binding (Fig. 4D).

Although omega-GST also has DHAR activity, the AsA-binding site of OsDHAR is distinct from that of hGSTO1-1^20^. The AsA-binding cleft in the hGSTO1-1 is located at the G-site generated by β3, β4, and α3. In detail, the bound AsA in omega-GST is stabilized by hydrogen-bonding interactions with the backbone of Ser86 and hydrophobic interactions with Lue71, Val72, and Phe34. In contrast, the bound AsA in OsDHAR is surrounded by α1, α5, and α7, forming the H-site^20^ (Supplemental Fig. S3 online). The bound AsA in OsDHAR interacts with Lys8 and Lys210 via hydrogen bonds and forms van der Waals interactions with Pro21, Phe104, and Trp207.

### Overall structure of GSH-bound OsDHAR

We also determined the structure of GSH-bound OsDHAR (C20S) at 1.9 Å to directly compare the binding site with that of the AsA-bound structure. Interestingly, GSH binds to a site similar to that in the AsA-bound structure but slightly away from Ser20 and towards the C-terminal domain (Fig. 3B). Notably, the γ-glutamyl-CA1 region of GSH interacts relatively weakly with OsDHAR and shows poor electron density. In comparison, the other region of GSH contains three hydrogen bonds and several hydrophobic interactions with OsDHAR as well as strong electron density. The carboxylic group of GSH forms a tight hydrogen bonding network with Lys8, His160, and Asp19. Among these, the side chain of Lys8 is involved in a water (W1)-mediated hydrogen bond with GSH (Fig. 4B).

Structural comparison of the GSH- and AsA-bound OsDHAR structures showed that the side chain of Trp207 was rotated slightly towards the CA3 atom of GSH to produce a hydrophobic interaction. Two other residues, Lys8 and Lys210, also displayed altered interactions and movements, as shown in Fig. 4D. The results show that the side chain of residue Lys8 undergoes a large movement and forms a W1-mediated hydrogen bond with GSH. During GSH entry, the Lys210 residue likely retreats from its position in the AsA-bound structure to make room for the longer bond formed with GSH.

**Figure 5:**
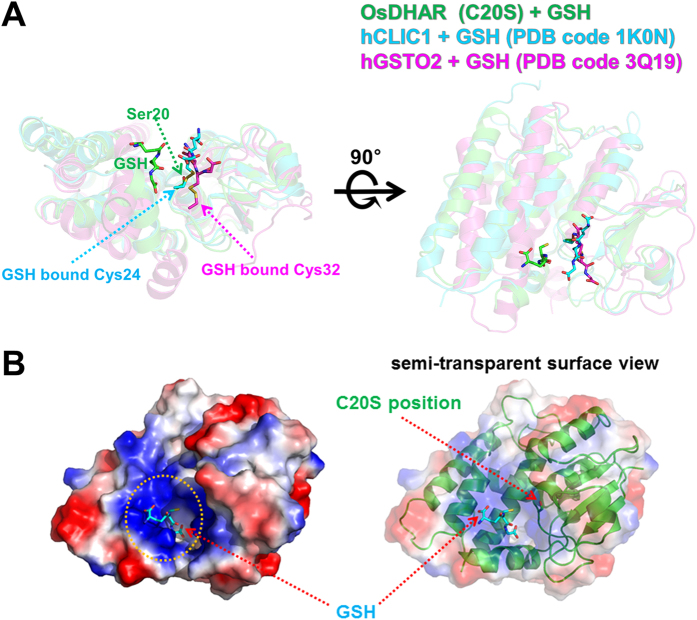
Comparison of the GSH-binding site of OsDHAR (C20S mutant) with CLIC1 and GSTO2. **(A)** Structural superposition of GSH-bound OsDHAR, hCLIC1 (PDB code 1K0N, cyan), and hGSTO2 (PDB code 3Q19, magenta). The orientation of the figure on the right is rotated 90° around the horizontal axis with respect to that on the left. The active sites of hCLIC1 and GSTO2 are shown with the covalently attached GSH at Cys24 and Cys32, respectively. Bound GSH molecules are shown as stick models. In contrast with hCLIC1 and GSTO2, GSH in OsDHAR is located in a different binding site. **(B)** Surface representation of OsDHAR with the bound GSH molecule shown as cyan sticks. The C20S position is indicated in a semi-transparent surface view (right panel).

The structures of hCLIC1 (PDB code 1K0N) and hGSTO2 (PDB code 3Q19) were previously determined and were shown to share approximately 20% sequence identity with OsDHAR (Fig. 2B)^12^,^17^,^20^. The two proteins have apparent DHAR activity and contain GSH, which appears to be covalently attached to the catalytic cysteine residue via a disulfide bond at the active site^11^,^20^. Structural alignment of the GSH-bound OsDHAR (C20S mutant), hCLIC1 (PDB code 1K0N; GSH is covalently linked at the Cys24 residue), and hGSTO2 (PDB code 3Q19; GSH is covalently linked at the Cys32 residue) allowed us to identify another GSH binding site in the OsDHAR structure. When the GSH binding site was identified, we initially predicted that our GSH-bound OsDHAR structure may represent a GSH binding site that existed prior to covalent bond formation with Cys20 (Fig. 5). However, further examination revealed that the GSH binding site is very similar to that in bacterial *Ochrobactrum anthropi* GST (OaGST) as an H-site binding^21^. Previous studies of human omega class GSTs and OaGST showed that there are two GSH binding sites, referred to as the G-site (GSH binding) and the H-site (hydrophobic binding)^18^,^20^,^21^ (Supplemental Fig. S4 online). The G-site is formed by highly conserved amino acid residues in the N-terminal domain, while the H-site is formed by less conserved hydrophobic residues in the C-terminal domain. Generally, GSH binding shows relatively strong affinity to the G-site (near the active cysteine residue) and weak affinity to the H-site in the case of WT proteins. In enzymes exhibiting DHAR activity, the first cysteine residue in the G-site motif is known to react with the sulphur atom in disulfide bonds within GSH mixed disulfide bridges, and then promotes thiol transfer. However, mutation of the active cysteine residue induces a significant loss of catalytic efficiency as well as GSH binding affinity at the G-site^19^,^20^,^21^. This also switches the preferential binding of GSH to the H-site. The GSH-bound OsDHAR structure also was determined using the C20S mutant protein, which showed that GSH bound at the H-site. Structural comparison of the GSH-bound OsDHAR and GSH-bound OaGST structures revealed that the binding position of GSH is very similar to the H-site binding, although the residues involved in the H-site binding of GSH are very different from each other. In the OaGST structure, the bound GSH molecule forms hydrophobic interactions with Phe112 and Trp164 and two hydrogen bonds between His105 and Ser109^21^. Moreover, structural superposition between GSH-bound OsDHAR and glutathione disulphide (GSSG)-bound hGSTO1-1 showed dramatic helix (α5, α6, and α9) movement in the C-terminal domain region of OsDHAR, which likely allows bound GSH molecules in the G- and H-sites to be sufficient close for GSSG formation (Supplemental Fig. S4 online)^18^. Furthermore, the rather long distance of 5.8 Å between the SG2 atom of GSH and the OG atom of Ser20 residue suggests that the H-site bound GSH reacts with G-site bound GSH rather than the active site cysteine residue.

### Overall structure of oxidized OsDHAR

**Figure 6:**
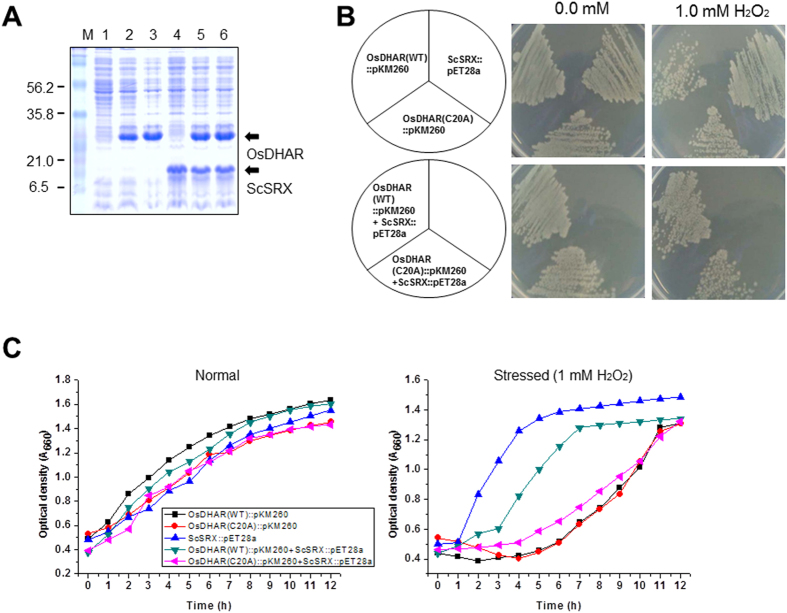
Gene expression and stress response in *E. coli* NiCo21(DE3) cells. These experiments were conducted as described in the *Supplemental Methods section*. **(A)** Protein expression analyzed by SDS-PAGE. M, protein marker; lane 1, cells without vector; lane 2, cells containing OsDHAR(WT)::pKM260 vector; lane 3, cells containing OsDHAR(C20A)::pKM260 vector; lane 4, cells containing ScSRX::pET28a vector; lane 5, cells containing OsDHAR(WT)::pKM260 plus ScSRX::pET28a vectors; lane 6, cells containing OsDHAR(C20A)::pKM260 plus ScSRX::pET28a vectors. **(B)** Stress response to streaking assay in the presence of 1 mM H_2_O_2_. Left panel, normal conditions; right panel, H_2_O_2_-treated conditions. **(C)** Growth kinetics in the presence of 1 mM H_2_O_2_. Square, cells with OsDHAR(WT)::pKM260; circle, cells with OsDHAR(C20A)::pKM260; upward triangle, cells with ScSRX::pET28a vector; downward triangle, cells with OsDHAR(WT)::pKM260 plus ScSRX::pET28a vectors; left triangle, cells with OsDHAR(C20A)::pKM260 plus ScSRX::pET28a vectors.

**Figure 7:**
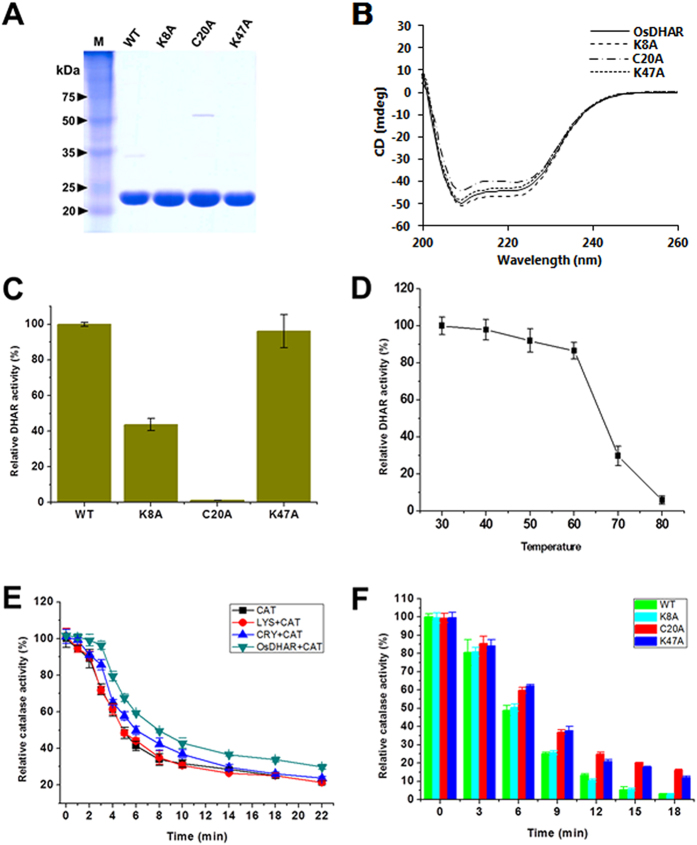
Enzymatic and chaperone-like activity of OsDHAR. **(A)** Purified OsDHAR proteins (WT and mutants) were separated by 12% SDS-PAGE gel and visualized using Coomassie blue staining to evaluate the purity prior to the activity assay. **(B)** Circular dichroism (CD) spectra of wild-type OsDHAR and mutants. The CD spectra of all mutants were similar to those of the wild-type OsDHAR, indicating that the overall conformation of the mutants was unaffected by the mutagenesis. **(C)** The DHAR activity of mutants was compared with wild-type OsDHAR. K8A and C20A mutants showed markedly decreased DHAR activity, whereas the DHAR activity of K47A was similar to that of the wild-type enzyme. **(D)** The effect of temperature on DHAR activity was measured using wild-type OsDHAR. **(E)** The anti-aggregation activity of OsDHAR was evaluated by using catalase (CAT). A general chaperone, crystallin (CRY) was also tested and was shown to have a clear protective effect on catalase. However, lysozyme (LYS) showed no protective effects on CAT. In this assay, OsDHAR showed the strongest anti-aggregation activity among the tested proteins. These data suggest that OsDHAR clearly exhibited chaperone-like activity. **(F)** The chaperone-like activities of wild-type and mutant OsDHAR were measured, and the results showed similar activity despite the mutations, suggesting that this chaperone-like activity was not correlated with enzymatic activity.

Initially, DHA-bound OsDHAR structural determination was attempted to examine the DHA binding site and the structural differences compared to the apo form. Although DHA was present in the crystallization solution and in the cryo-buffer, no electron density for DHA was observed in the electron density maps, except for a large spherical region of electron density surrounding Cys20 (Fig. 3C). Given that the cysteine residue was oxidized in the presence of DHA, this density was modelled as cysteine sulfonic acid (Supplemental Fig. S5 online). To examine whether sulfonylation of the cysteine residues affects the enzyme activity of OsDHAR, we grew *Escherichia coli* cells harbouring single genes [*OsDHAR* (WT), *OsDHAR* (C20A), and *ScSRX*] and dual genes [*OsDHAR* (WT) + *ScSRX* and *OsDHAR* (C20A) + *ScSRX*]. The expression of each gene was confirmed by SDS-PAGE (Fig. 6A). Cells co-expressing *OsDHAR* and *ScSRX* rapidly recovered under H_2_O_2_-induced oxidative stress compared to cells expressing *OsDHAR* (WT), *OsDHAR* (C20A), and *OsDHAR*(C20A) + *ScSRX* (Fig. 6B,C). The stress adaptation ability improved because of the modification of cysteine residues (Supplemental Fig. S6 online). The cysteine residues (Cys20 and Cys106) of OsDHAR were sulfonylated in the absence of sulfiredoxin (*ScSRX*) under oxidative conditions (Supplemental Fig. S7 and S8 online), whereas Cys20 was sulfenylated in the presence of *ScSRX* under the same conditions (Supplemental Fig. S9 online). Oxidation of cysteine residues can result in a wide range of redox-based, post-translational modifications, including S-nitrosylation, S-glutathionylation, and sulfinic acid, sulfenic acid, and disulfide formation^22^,^23^, which can generate conformational changes in protein structure and subsequent modulation of protein activity^24^. Importantly, such modifications precisely regulate protein structure and function^22^,^23^. In contrast to the reversibly hyperoxidized cysteine sulfenylated proteins, irreversibly hyperoxidized sulfonylated cysteine is generally thought to be an impaired dead end product (e.g., inactivated protein) formed through protein aggregation^25^. Sulfinic acid and sulfenic acid can mainly be reduced by GSH or thioredoxin and sulfiredoxin in biological systems, respectively. In addition, putative plant Srx proteins capable of reducing the sulfinic acid moiety through an unusual ATP-dependent mechanism as a repair enzyme^26^ showed significant identities to their orthologs from yeast and humans and exhibited conserved signature sequences and residues essential for catalysis^27^. Srx proteins from *Arabidopsis* (*AtSRX*) and rice (*OsSRX*) complemented the functional deficiency of Srx in *SRX1* deletion yeast cells^27^. Taken together, sulfiredoxin plays a critical role in oxidative stress tolerance in *E. coli* and plant cells by preventing the formation of hyperoxidized cysteine sulfonylation in OsDHAR. Numerous cysteine-based redox switches have been found to underpin many different signalling systems and regulate physiological outputs across kingdoms^22^,^23^. Notably, the side chain of Lys8 showed a large movement in the structural superposition of reduced and oxidized OsDHAR structures and formed a hydrogen bond (distance = 3.1 Å) with the side-chain oxygen of Cys20-sulfonic acid (Figs. 3C and 4C). As described above, the Lys8 residue is also involved in the direct binding to AsA and the W1-mediated hydrogen bond with GSH. Thus, we adopted a mutational approach to evaluate and confirm the role of the Lys8 residue in the enzymatic reaction. As expected, the K8A mutation significantly reduced the enzymatic activity of OsDHAR (Fig. 7A–C), indicating that the Lys8 residue is critical for the enzymatic activity of OsDHAR.

### Chaperone-like activity of OsDHAR

In our lab, we found that OsDHAR exhibited both DHAR and chaperone-like activity *in vitro*. For this experiment, the temperature was maintained at 60 °C, as OsDHAR and catalase (CAT) aggregate at this temperature over time (Fig. 7D,E). To investigate whether OsDHAR is capable of protecting other enzymes by reducing an aggregation-induced loss of function, we tested the enzymatic activity of CAT before and after exposure to 60 °C for 22 min in the presence or absence of OsDHAR. In the presence of OsDHAR, there was a significant increase in CAT enzymatic activity compared to in the presence of LYS or CRY, which were used as negative and positive controls, respectively (Fig. 7E). Taken together, these results indicate that OsDHAR has potent chaperone-like properties and is capable of protecting proteins from precipitation and heat-induced loss of enzymatic activity, suggesting that OsDHAR is a potent inhibitor of stress-induced protein aggregation. As shown in Fig. 7F, the oxidation of OsDHAR did not significantly alter the chaperone-like activity of OsDHAR, and the three mutants displayed similar chaperone-like activities. These results indicate that DHAR enzymatic activity is not correlated with chaperone-like activity. Notably, the three-dimensional structure indicates that OsDHAR contains a broad, uncharged cleft between the α2-helix and the α3-helix as the putative binding site for non-native protein folding intermediates for its chaperone-like activity.

### Proposed mechanism of AsA regeneration by OsDHAR

**Figure 8:**
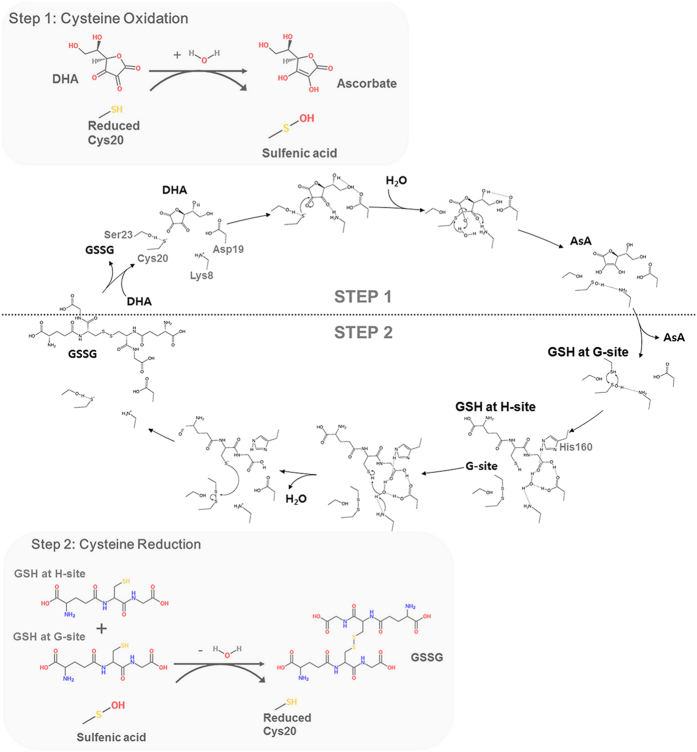
Proposed enzymatic reaction scheme for OsDHAR. The proposed catalytic mechanism of OsDHAR involves a water molecule as a hydrogen donor for the DHA-reduction step and for GSH stabilization, forming hydrogen bonds with Lys8, His160, and Asp19 residues of OsDHAR.

Figure 8 is a schematic representation of our current hypothesis, showing an overview of the catalysis for OsDHAR accompanying the active site cysteine oxidation and reduction. OsDHAR catalyses the GSH-dependent reduction of DHA to recycle AsA in a two-step reaction: the first step is the cysteine oxidation reaction and second step is the cysteine reduction reaction. In the first step of the reaction, a DHA ligand molecule binds to OsDHAR with a reduced catalytic Cys20 residue. In this step, the Lys8 residue may have critical role in recognizing the DHA. The data presented in Fig. 4 support this prediction. In contrast, the Lys8 residue had a different conformation in the oxidized state of Cys20 as shown in our oxidized OsDHAR structure. Thus, Lys8 movement would also allow the product, AsA, to be released. DHA reduction involves nucleophilic attack by the Cys20 and the formation of the cysteine sulfenic acid. For this reaction, Ser23 may stabilize the ionized cysteine residue. In the apo-OsDHAR structure, the OG atom of Ser23 is 3.4 Å away from the SG atom of Cys20, which is an acceptable hydrogen bonding distance considering the rotational movement of the side chains of Ser23 and Cys20. Notably, the Lys8 residue is highly conserved across all DHAR and CLIC proteins, but human omega GSTs contain a proline residue at this corresponding position, suggesting that omega GSTs may have different cysteine oxidation strategies^20^ (Fig. 2B). To further clarify the role of the Lys8 residue in the function of OsDHAR, we examined the effects of substitution of Lys to Ala at this position. As shown in Fig. 7, the K8A mutant protein showed significantly reduced DHAR activity. Next, the reactive cysteine residue may attack the 2′ oxygen of DHA and produce AsA, consuming one water molecule. Next, the AsA is displaced by GSH binding for the second reaction. Unfortunately, there is an experimental limitation to closely investigating the first step of the reaction of OsDHAR. It is very difficult to observe the DHA-bound OsDHAR complex structure because DHA is very unstable and easily degraded in solution. Thus, we attempted to solve the complex structure with a more stable product compound, AsA, using active site mutant protein (C20S).

In the second reaction, the active site cysteine reduction and regeneration of OsDHAR is initiated by binding of the first GSH molecule at the G-site followed by the second GSH binding at the H-site. Based on our GSH-bound OsDHAR structure and other studies from omega class GSTs and bacterial OaGST, the regeneration of OsDHAR may proceed via a disulfide bond link between the G-site GSH and active site cysteine residue. The sulfenic acid at Cys20 reacts with G-site bound GSH to generate a mixed disulfide (enzyme-S-S-G). Sulfenic acid is an unstable and reactive molecule; thus, it readily reacts with the SH group of G-site bound GSH to form a disulfide linkage^22^,^23^. Subsequently, the second GSH molecule can bind to the H-site and the H-site bound GSH is deprotonated into the GS^−^ form. In the GSH-bound OsDHAR structure, a well-ordered water molecule was found near the bound GSH molecule, forming hydrogen bonds with the Lys8, Asp19, His160 residues. The water appears to be positioned to pull off the proton from the sulphur atom of GSH. Thus, we hypothesize that the water molecule can act as a possible nucleophile to attack the H-site bound GSH and generate GS^−^. Notably, the inter-atomic S-O distance between the H-site bound GSH and water molecule was 3.6 Å, which is slightly greater than the expected hydrogen bond length as a nucleophile. However, this reaction occurs only when both the H-site and G-site are simultaneously bound to GSH, and a slight conformational change may allow ideal coordination geometry of the water molecule in the active site. Apparently, structural comparison of the GSH-bound form of OsDHAR (enzyme-S-S-G) and GSSG-bound human omega GST showed a large conformation change and active site rearrangement around H-site region. Notably, the mechanism of human GSTO1-1 may be that the backbone amide group of Phe34 donates a hydrogen bond to the sulphur atom of the H-site bound GSH^18^. However, the corresponding Phe22 residue in OsDHAR is located too far away from the H-site bound GSH (distance between the NH of Phe22 and S of GSH was 6.5 Å) for an interaction to occur, even after the conformational change^20^. This result also indicates that OsDHAR has a different enzymatic reaction mechanism with omega GSTs^28^. Next, the GSH linked with the active site cysteine residue of OsDHAR (enzyme-S-S-G) is removed by nucleophilic attack of the H-site bound GS^−^. As a result, a reduced cysteine residue is produced and one molecule of GSSG is released. Taken together, the reaction scheme described in Fig. 8 suggests a ping-pong kinetic mechanism of OsDHAR, with one ping-pong reaction at the cysteine active site region and one ordered sequential binding site for GSH at H-site^29^.

In conclusion, our results suggest that OsDHAR is involved in AsA recycling as well as the inhibition of protein aggregation. Although the major function of OsDHAR may be to reduce DHA to protect against ROS, this protein may also serve to protect other proteins in response to a broad variety of stress conditions. These results represent the most detailed description of the catalytic reduction mechanism of OsDHAR at atomic resolution and are fully consistent with all available biochemical data (Fig. 8). No three-dimensional structure of a DHAR has been reported. Here, we present the first crystal structure of OsDHAR. In addition, the residues involved in ascorbate and GSH recognition have been determined, and a structural comparison with CLIC1 and other GST enzymes was discussed. Finally, the results presented here provide useful information for understanding the catalytic mechanism and structure-function relationship of OsDHAR.

## Methods

### Expression and purification of recombinant OsDHAR

Recombinant OsDHAR was expressed in *Escherichia coli* NiCo21(DE3), purified, and crystallized as described previously^30^. Briefly, the gene encoding the OsDHAR sequence (residues 2–203) was amplified by PCR using *Ex Taq* polymerase (TaKaRa Bio Inc., Shiga, Japan) with forward and reverse primers for a cDNA template (Table S1). The PCR products were digested with *Nco*I and *Bam*HI and ligated into the pKM260 expression vector (EUROSCARF, Frankfurt, Germany), which resulted in the N-terminal addition of a hexahistidine tag and linker with the sequence MHHHHHHASENLYFQGAM. The ligated plasmids (pKM260::OsDHAR) were sequenced using the T7 promoter and terminator primers, and all sequences were verified. The plasmids were then transformed into CaCl_2_-competent *E. coli* NiCo21(DE3) cells at 42 °C by heat-shock. The cells were incubated overnight at 37 °C on Luria-Bertani-ampicillin agar plates, and transformants were selected and grown overnight in 50 mL of LB medium containing 100 μg/mL of ampicillin at 37 °C. Next, 40 mL of seed culture grown overnight was added to 4 L of LB broth and incubated at 37 °C until it reached an optical density of 0.6 as measured at 600 nm (*A*_600_). Protein expression was induced with 0.2 mM IPTG for 20 h at 20 °C with shaking. After harvesting the cells by centrifugation at 4,000 ×*g* for 30 min, the cells were resuspended in lysis buffer (50 mM sodium phosphate, pH 8.0, 300 mM NaCl, 10 mM imidazole) and lysed using a sonication for 1 h with a 10-s pulse mode (10 s on and 10 s off) on ice. OsDHAR was purified by Ni-NTA affinity chromatography [5 mL HisTrap FF column (GE Healthcare, Little Chalfont, UK) on an AKTA FPLC system] and eluted with a step gradient of 250 mM imidazole. The fractions containing OsDHAR protein were pooled and concentrated using Amicon Ultra-15 Centrifugal Filters (Ultracel-10 K; Merck Millipore Ltd., County Cork, Ireland). Next, the proteins were purified by gel filtration in a Superdex 200 column (Amersham Biosciences Inc., Piscataway, NJ, USA) equilibrated in 20 mM Tris-HCl (pH 8.0) and 150 mM NaCl. The fractions containing OsDHAR protein were pooled and concentrated to 17 mg/mL.

The construction of the mutant proteins was achieved by site-directed PCR mutagenesis using mutagenic primers (Table S1). The pKM260::OsDHAR plasmid was used as a template for site-directed mutagenesis to introduce the K8A, C20A, C20S, and K47A point mutations into the *OsDHAR* gene. Mutagenic primer sequences were designed (Table S1), and the DNA constructs were amplified by PCR. Following mutagenesis, the plasmid template was digested using the *Dpn*I restriction enzyme to eliminate template plasmids and the plasmid was then transformed into competent *E. coli*(DH5α). Each mutant was confirmed by DNA sequencing. Individual mutant clones were transformed into *E. coli* strain NiCo21(DE3) or BL21(DE3) for protein expression. All mutant proteins were expressed and purified using a procedure similar to that used for the wild-type (WT) OsDHAR.

### Crystallization and X-ray diffraction data collection

The crystallization and preliminary X-ray diffraction data analysis of the apo-OsDHAR were performed as described previously^30^. Briefly, the crystals were produced in a solution of 0.15 M potassium bromide and 30% (w/v) PEG MME 2000 using the hanging drop vapour diffusion method in 24-well plates. The drops contained 1 μL of protein solution and 1 μL of reservoir solution and were equilibrated against 500 μL of reservoir solution. The plate-shaped crystals grew to maximum dimensions of 0.2 × 0.2 × 0.05 mm after 3 days at 298 K and were immersed in Paratone-N and dragged back and forth for 1 min before being flash-frozen in a stream of liquid nitrogen at 100 K for X-ray diffraction. A complete data set at 1.9 Å resolution was collected at beamline BL-5C of the Pohang Accelerator Laboratory (Pohang, Korea). The HKL-2000 package (HKL Research Inc., Charlottesville, VA, USA) was used to index, integrate, and scale the X-ray diffraction data (Table S2). A complete data set at 1.9 Å resolution was collected.

The oxidized-OsDHAR data were collected unintentionally. We attempted to collect data for the structure of the complex with DHA (3 mM) by the soaking method using the apo-OsDHAR crystals. After testing various incubation times and concentrations of DHA, a structure with oxidized Cys20 was collected rather than the complex formed with DHA. We assumed that DHA oxidizes Cys20 and that the oxidation would affect the affinity of ascorbate. Diffraction data were collected on an ADSC Quantum 4R CCD detector at beamline BL-7A of the Pohang Accelerator Laboratory. Subsequent indexing, integration, and scaling of these data were conducted using the HKL-2000 package. Data collection and processing statistics are provided in Table S2. Because Cys20 oxidizes naturally as well as artificially, the C20S mutant was used for structural determination of the complexes.

The structure of the complex containing ascorbate or GSH was solved by the ligand soaking method. The OsDHAR-C20S crystals were grown at 293 K using the vapour diffusion method. Drops (2 μL) consisting of a 1:1 ratio of C20A mutant OsDHAR protein (21 mg/mL in 20 mM Tris-HCl, pH 8.0, and 150 mM NaCl) and crystallization solution (100 mM Bis-Tris HCl, pH 6.1–6.8, 28% (w/v) PEG 3350, and 10 mM CaCl_2_) were equilibrated against 0.5 mL of the same solution. Either 0.5 μL of 3 mM AsA or 5 mM GSH dissolved in buffer (10 mM Bis-Tris, pH 6.5, 30% (w/v) PEG 3350, and 1 mM DTT) was added to each drop of fully grown crystals and maintained for 72 h before the diffraction test. The crystal was directly mounted without any cryoprotectants. The space group of OsDHAR crystals was P2_1_ and contained one molecule in the asymmetric unit. Diffraction data were collected up to 1.7 Å resolution for the complex crystals. Diffraction data were collected at the Pohang Accelerator Laboratory (PAL, beamline BL-7A) and were processed and scaled using the HKL-2000 package^31^ (Table S2).

### Structural determination and refinement

Structural determination of reduced OsDHAR was performed by the molecular replacement method with the MOLREP program by using a homology model of OsDHAR generated by the SWISS-MODEL server^32^ and the coordinates of human CLIC1 as the template structure^12^. The highest peak yielded a contrast of 1.63. Rigid body refinement of reduced OsDHAR was performed using the REFMAC5 program by placing the best molecular replacement solution. The OsDHAR sequence was then manually built into the electron density map using the COOT program, and restrained refinement was performed using the REFMAC5 program^32^,^33^. The resulting model was improved by manual inspection and rebuilding in COOT and refined in REFMAC5 or Phenix^33^,^34^,^35^.

The structures of the AsA-OsDHAR and GSH-OsDHAR complexes were determined by molecular replacement using the MOLREP program. The refined apo-OsDHAR structure was used as a search model. Subsequently, structural modelling and refinement were performed using COOT and REFMAC5^33^,^34^. All residues of OsDHAR, including the AsA molecule, could be traced in the electron density map and exhibited good stereochemistry. The structure of oxidized OsDHAR was solved and refined using similar protocols. The geometries of the refined structures were analyzed and validated using MolProbity^36^. The structural refinement statistics are summarized in Table S2.

### Reductase enzymatic activity

The reductase activities of the WT and mutant (K8A, C20A, and K47A) proteins were measured spectrophotometrically by recording the increase in absorbance at 260 nm using a UV-VIS spectrophotometer (UV-1800, Shimadzu, Kyoto, Japan) as described previously^37^. The reaction mixture contained 50 mM sodium phosphate, 0.5 mM EDTA (pH 7.0), 1 mM GSH, and 0.5 mM DHA in a total volume of 1 mL. DHA and GSH were prepared immediately before use. The reaction was initiated by the addition of DHA and was recorded for 1 min at 30 °C with a 1-cm path length. The reaction rate was calculated by using an extinction coefficient of 14.7 mM/cm. The linear region for enzymatic activity was determined based on the coefficient of determination (R^2^ > 95).

### Circular dichroism analysis

The overall structures of the OsDHAR and OsDHAR mutants were predicted by using circular dichroism analysis to examine mutational changes in the OsDHAR mutants. Far-UV CD was performed on a Chirascan CD spectrometer (Applied Photophysics Ltd., Leatherhead, UK) between 200 and 260 nm by using a 1-mm path length cell at 293 K. Five scans were recorded; baseline spectra (buffer: 20 mM Tris pH 7.0, 150 mM NaCl) were subtracted from the averaged spectrum followed by smoothing of the data.

### Chaperone-like activity assays

Chaperone-like activity was measured to investigate whether OsDHAR could protect other enzymes from heat-induced loss of activity. Lysozyme (LYS; 0.25 mg/mL), α-crystallin (CRY; 0.25 mg/mL; Sigma-Aldrich, Saint Louis, MO, USA), or OsDHAR (0.25 mg/mL) was added to catalase (CAT; 0.1 mg/mL) in a reaction mixture containing 10 mM phosphate buffer (pH 7.0), which was then heated at 60 °C for 22 min. CRY and LYS were used as positive and negative controls, respectively. To examine the effects of mutations on chaperone-like activity, OsDHAR WT, K8A, C20A, or K47A (0.2 mg/mL) was added to CAT (0.1 mg/mL) in the same reaction mixture and then heated at 60 °C for 22 min. A sample aliquot was removed at each of the indicated times. CAT activity was measured at 240 nm for 0.5–1 min in 1 mL of the reaction mixture containing 10 mM phosphate buffer (pH 7.0), 15 mM H_2_O_2_, and CAT (10 μg). Enzymatic activity of the unheated CAT solution alone was defined as 100%.

### Statistical analysis

All experiments were performed in at least triplicate, and the results are expressed as the mean and standard deviation (SD). Relative data are presented relative to the WT, which was defined as 100%.

## Additional Information

**How to cite this article**: Do, H. *et al*. Structural understanding of the recycling of oxidized ascorbate by dehydroascorbate reductase (OsDHAR) from *Oryza sativa* L. *japonica. Sci. Rep.* **6**, 19498; doi: 10.1038/srep19498 (2016).

## Supplementary Material

Supplementary Information

## Figures and Tables

**Figure 1:**
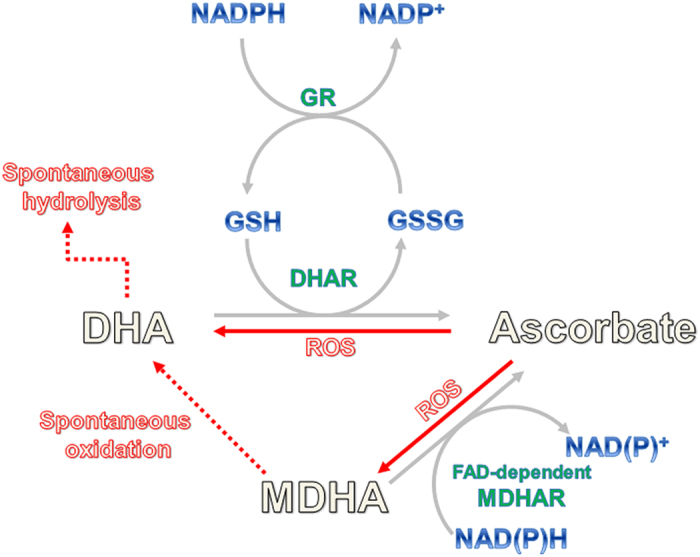
Ascorbate is oxidized by ROS and converted into two different oxidized forms, dehydroascorbate (DHA) and monodehydroascorbate (MDHA). The ascorbate (AsA) recycling is catalysed by a set of three enzymes: glutathione (GSH)-dependent dehydroascorbate reductase (DHAR), FAD-dependent monodehydroascorbate reductase (MDHAR), and glutathione reductase (GR).
