# Supplementary material for: Structural understanding of the recycling of oxidized ascorbate by dehydroascorbate reductase (OsDHAR) from Oryza sativa L. japonica
Source: Sci Rep. 2016 Jan 18;6:19498. doi: 10.1038/srep19498 (PMC4726096; doi:10.1038/srep19498)
Supplement: Supplementary Information [file srep19498-s1.doc]

**SUPPLEMENTARY INFORMATION**

**Structural Understanding of the Recycling of Oxidized Ascorbate by Dehydroascorbate Reductase (OsDHAR) from *Oryza sativa* L. *japonica***

Hackwon Do^1,5^, Il-Sup Kim^2,5^, Byoung Wook Jeon^1,5^, Chang Woo Lee^1,3^, Ae Kyung Park^1^, Ah Ram Wi^1^, Seung Chul Shin^1^, Hyun Park^1,3^, Young-Saeng Kim^4^, Ho-Sung Yoon^2*^, Han-Woo Kim^1,3*^, and Jun Hyuck Lee^1,3*^

^1^Division of Polar Life Sciences, Korea Polar Research Institute, Incheon 406-840, Republic of Korea

^2^Department of Biology, Kyungpook National University, Daegu 702-701, Republic of Korea

^3^Department of Polar Sciences, University of Science and Technology, Incheon 406-840, Republic of Korea

^4^Division of Biological Sciences, University of California San Diego, La Jolla, California 92093-0116, USA

^5^H.D., I-S.K., and B.W.J. equally contributed to this work.

^*^To whom correspondence should be addressed:

Dr. Ho-Sung Yoon, Department of Biology, College of Natural Sciences, Kyungpook National University, Daegu 702-701, Republic of Korea; Tel: +82-53-950-5348; Fax: +82-53-953-3066; E-mail: hsy@knu.ac.kr

Dr. Han-Woo Kim, Division of Polar Life Sciences, Korea Polar Research Institute, Incheon 406-840, Republic of Korea, Tel: +82-32-760-5526; Fax: +82-32-760-5509; E-mail: hwkim@kopri.re.kr

Dr. Jun Hyuck Lee, Division of Polar Life Sciences, Korea Polar Research Institute, Incheon 406-840, Republic of Korea; Tel: +82-32-760-5555; Fax: +82-32-760-5509; E-mail: junhyucklee@kopri.re.kr

**Supplemental Figure S1.** Purified OsDHAR is monomer in solution and was not affected by oxidation or DHA binding. Purified OsDHAR with 1 mM DTT (black), OsDHAR with 10 mM H_2_O_2_ (brown), and OsDHAR with 2 mM DHA (blue) samples were analyzed by size exclusion chromatography on a Superdex 200 10/300 GL column and showed estimated molecular weights of 26.0, 27.0, and 27.7 kDa, respectively. The miner peak from OsDHAR with 10 mM H_2_O_2_ (brown) sample was not the OsDHAR protein. Probably, it may be aggregated impurities. The β-amylase (200 kDa, V_e_ = 11.95 mL), albumin (66 kDa, V_e_ = 14.31 mL), carbonic anhydrase (29 kDa, V_e_ = 16.70 mL), and cytochrome C (12.4 kDa, V_e_ = 17.97 mL) proteins were used for standard curve and molecular weight calculations.

**Supplemental Figure S2.** hGSTO1-1 and hCLIC1 form dimers with different subunit interfaces. (A) Ribbon diagram of apo-OsDHAR structure. OsDHAR was consistently observed to be a monomeric protein, regardless of oxidation or ligand binding. (B) Structural superposition of hGSTO1-1 dimer (PDB code 4IS0) onto the apo-OsDHAR structure. For clarity, bound ligands were removed from the hGSTO1-1 structure. (C) Structural superposition of the hCLIC1 dimer (PDB code 1RK4) onto the apo-OsDHAR structure. The orientation of the OsDHAR structure is the same in all panels.

**Supplemental Figure S3.** Comparison of AsA-binding sites of OsDHAR and human GSTO1-1. (A) Ribbon diagram of AsA-bound OsDHAR structure. The bound AsA molecule is shown as a stick model and highlighted in green. (B) Ribbon diagram of AsA-bound human GSTO1-1 structure (PDB code 3VLN). The bound AsA molecule is shown as a stick model and highlighted in yellow. (C) The bound AsA and the interacting residues in OsDHAR are represented in a stick model. (D) The bound AsA and the interacting residues in human GSTO1-1 are represented in a stick model.

**Supplemental Figure S4.** Structural comparison of GSH-bound OsDHAR, GSH-bound OaGST, and GSSG-bound human GSTO1-1. (A) Ribbon diagram of GSH-bound OsDHAR structure. The bound GSH molecule is shown as a stick model and highlighted in green. (B) Ribbon diagram of GSH-bound OaGST structure (PDB code 2PVQ). The bound GSH molecule is shown as a stick model and highlighted in green. (C) Ribbon diagram of GSSG-bound hGSTO1-1 structure (PDB code 4IS0). The bound GSSG molecule is shown as a stick model and highlighted in green. (D) The bound GSH and interacting residues in OaGST structure are shown as stick models. (E) Ribbon diagram showing superposition of the GSH-bound OsDHAR (green) and GSSG-bound hGSTO1-1 structure (cyan). The bound GSH and GSSG are shown as stick models in green and blue, respectively.

**Supplemental Figure S5.** Electron density surrounding Cys20 in the oxidized OsDHAR structure. (A) The Fo-Fc omit map (green, contoured at 2.0 σ) was calculated using the thiol model of Cys20. (B) The 2Fo-Fc map (grey, contoured at 2.0 σ) was shown after final modelling of the oxidized cysteine. The positive electron density (green) observed in the Fo-Fc omit map disappeared because of the oxygen atoms of the sulfonic acid.

**MATERIALS AND METHODS**

**Plasmid construction and expression of recombinant *ScSRX***

Genomic DNA was isolated from stationary phase cells of *Saccharomyces cerevisiae* S288C using an RNA/DNA Mini Kit (Qiagen, Hilden, Germany) according to the manufacturer’s instructions. The gene encoding sulfiredoxin (*ScSRX*; YKL086W, or NP_012837) was amplified from the *S. cerevisiae* genomic DNA by PCR using *ExTaq* polymerase (Takara Bio Inc., Shiga, Japan) with 5′-TGCCGCGCGGCAGCCATATGCACTACAAAGCAACAGTGT-3′ and 5′- TGGTGGTGGTGGTGCTCGAGTCATTCGATGTCGAGACTGC-3′ as the sense and antisense primers, respectively. Each restriction site is underlined. The PCR product was cloned into the *Nde*I and *Xho*I sites of pET28a(+) (Novagen, Darmsdast, Germany). Gene introduction was performed using the one-step sequence- and ligation-independent cloning method^1^. The cloned plasmid was transformed into competent *Escherichia coli* NiCo21(DE3) cells (New England BioLabs Inc., Ipswich, MA, USA) using the modified CaCl_2_-mediated method^2^. Positive transformants were grown on LB agar plates supplemented with 50 μg/mL of kanamycin (KAN) for 24 h at 37°C. Plasmid DNA was then isolated with a plasmid isolation kit (ELPIS Bio., Daejeon, Korea) according to the manufacturer’s protocols. The cloned plasmid was sequenced using the T7 primer set to confirm that no PCR-induced mutations had been introduced. The *E. coli* strain containing the correctly transformed plasmid was used for subsequent experiments. For ScSRX protein expression, *E. coli* NiCo21(DE3) cells harbouring the ScSRX::pET28a(+) plasmid were grown in LB medium supplemented with 50 μg/mL of KAN with vigorous shaking (200 rpm) at 37°C. When the culture reached the mid-log phase (A_660_ = 0.4), **IPTG was added at a final concentration of 0.2 mM, and the culture was incubated for another 3 h at** 37°C. The cells were then harvested by centrifugation at 4,000 × *g* for 20 min **at** 4°C and washed once with cold PBS. The resulting cell pellet was resuspended in lysis buffer containing 20 mM Tris-HCl, pH 8.0, 0.3 M NaCl, 10 mM imidazole, 1 mM PMSF, and an EDTA-free protease inhibitor cocktail (Roche Applied Science, Mannheim, Germany). Sonication was performed using a Sonic Dismembrator 550 (Fisher Scientific, Waltham, MA, USA) that was equipped with a micro tip (tip diameter of 3 mm) at a frequency of 20 kHz. The cells were intermittently sonicated on ice for 10 s followed by 10 s of cooling. The total sonication time was approximately 2.5 min. The homogenate was subsequently centrifuged at 13,000 × *g* for 30 min at 4ºC to remove cell debris. The protein concentrations of the cleared crude extracts were then determined using Protein Dye Reagent (Bio-Rad, Hercules, CA, USA), after which 20 μg of protein was subjected to 15% SDS-PAGE, run at 50 V, stained with CBB R-250, and then destained.

**Growth kinetics and streaking assay in transgenic *E. coli***

Mid-log phase *E. coli* cells (A_660_ = 0.4) grown in LB broth containing antibiotics (50 μg/mL KAN or 100 μg/mL ampicillin) and 10 mM sodium ascorbate were treated with 0.2 mM IPTG and cultured for an additional 1 h at 37°C with shaking (200 rpm). To remove residual ascorbate, *E. coli* cells were collected by centrifugation (4,000 × *g*, 20 min, 4°C), washed once with deionized distilled water, and resuspended in fresh LB broth medium containing 0.2 mM IPTG and 1 mM H_2_O_2_. Growth kinetics were monitored by measuring the optical density at 660 nm at 1 h intervals for 12 h. For the streaking assay, mid-log phase *E. coli* cells (A_660_ = 0.4) grown in LB broth containing antibiotics (50 μg/mL KAN or 100 μg/mL ampicillin) and 10 mM sodium ascorbate were treated with 0.2 mM IPTG and cultured for an additional 3 h at 37°C with shaking (200 rpm). The *E*. *coli* cells were then streaked onto LB agar plates supplemented with 1.0 mM H_2_O_2_, incubated for 24 h at 37°C, and then photographed.

**Cysteine modification**

The intact mass was measured by direct infusion into a liquid chromatography tandem mass spectrometer (LC-MS/MS) using a Surveyor nano-flow system connected to a 7-Tesla Finnigan LTQ-FT mass spectrometer (ThermoFischer Scientific, Waltham, MA, USA) equipped with a nano-electrospray ion source at the Korea Basic Science Institute. The mass spectra were deconvoluted using the ProMass Deconvolution software (ThermoFisher Scientific). To identify redox-active cysteine residues, OsDHAR was oxidized with H_2_O_2_. All free thiols were blocked with excess iodoacetamide for 10 min before tryptic digestion. The peptides were analyzed by LC-MS/MS^3^. The analytes were dissolved in acetonitrile/water (50:50, v/v) and loaded into preopened “medium” borosilicate spray capillaries for off-line nanoelectrospray. A potential of 1000 V was applied to the loaded glass capillary tip^4,5^. Electrospray ionization mass spectra were collected for 1 min, and the protein masses were calculated by deconvolving multiple charged ions with *m*/*z* values and corresponding total charges using the PERCOLATOR program within the Proteome Discoverer software (Thermo Fischer Scientific) and then manually validated. The considered dynamic modifications on cysteine residues were +138.0 Da for sulfenic acid, +32.0 Da for sulfinic acid, +48.0 Da for sulfonic acid, and +57.0 Da for carbamidomethyl modifications^3,6^.

**Statistical analysis**

The results of the growth kinetics and streaking assays were representative of at least 2 independent experiments conducted under identical conditions.

**REFERENCES**

1.Jeong, J. Y. *et al*. One-step sequence- and ligation-independent cloning as a rapid and versatile cloning method for functional genomics studies. *Appl. Environ. Microbiol.* **78**, 5440–5443 (2012).

2. Chung, C. T., Niemela, S. L. & Miller, R. H. One-step preparation of competent *Escherichia coli*: transformation and storage of bacterial cells in the same solution. *Proc. Natl. Acad. Sci. USA* **86**, 2172–2175 (1989).

3. [Waszczak, C](http://www.ncbi.nlm.nih.gov/pubmed/?term=Waszczak C%5BAuthor%5D&cauthor=true&cauthor_uid=25049418). *et al*. Sulfenome mining in *Arabidopsis thaliana*. *Proc. Natl. Acad. Sci. USA* **111,** 11545–11550 (2014).

4. Lim, J. C. *et al*. Irreversible oxidation of the active-site cysteine of peroxiredoxin to cysteine sulfonic acid for enhanced molecular chaperone activity. *J. Biol. Chem.* **283**, 28873–28880 (2008).

5. Pyr Dit Ruys, S. *et al*. Identification of autophosphorylation sites in eukaryotic elongation factor-2 kinase. *Biochem. J.* **442**, 681–692 (2012).

6. Kim, H. J., Ha, S., Lee, H. Y. & Lee, K. J. ROSics: chemistry and proteomics of cysteine modifications in redox biology. *Mass Spectrom. Rev.* **34**, 184–208 (2015).

**Supplemental Figure S6.** Modification overview of cysteine residues (Cys20 and Cys106) of OsDHAR in the presence of H_2_O_2_ using LC-MS/MS. Sulfenylation, sulfinylation, and sulfonylation of Cys20 **(A)** and Cys106 **(B)**.

**Supplemental Figure S7.** Detection of sulfonylation of OsDHAR Cys20 in the presence of H_2_O_2_ using LC-MS/MS.

**Supplemental Figure S8.** Detection of sulfonylation of OsDHAR Cys106 in the presence of H_2_O_2_ using LC-MS/MS.

**Supplemental Figure S9.** Sulfinylation identification of OsDHAR Cys20 following *ScSRX* expression in the presence of H_2_O_2_ using LC-MS/MS.

Table S1. Primers used for the amplification of wild-type and mutant OsDHAR genes

| Gene products | Forward primer | Reverse primer |
| --- | --- | --- |
| Wild-type | 5′-tcgccgccatgggcgtggaggtgtgcg-3′ | 5′-ccaagcaagggatcctgcaggctgcatctccattattc-3′ |
| K8A | 5′-ggtgtgcgtcgcggccgccgtcggccacccgg-3′ | 5′-gacggcggccgcgacgcacacctccacgcccatggc-3′ |
| C20A | 5′-ctcggcgacgccccattctcgcagagggtgctgct-3′ | 5′-cgagaatggggcgtcgccgagcgtgtccgggtgg-3′ |
| C20S | 5′-ataacccttgccatcgccggtgcctcgggtgct-3′ | 5′-agcacccgaggcaccggcgatggcaagggttat-3′ |
| K47A | 5′-cgtccagaacgcccccgactggtttctgaagatcagccc-3′ | 5′-accagtcgggggcgttctggacgtcgatgagcttca-3′ |

Table S2. Data collection and refinement statistics

| Data set | Apo OsDHAR | OsDHAR + Ascorbate | OsDHAR + GSH | Oxidized OsDHAR |
| --- | --- | --- | --- | --- |
| X-ray source  Space group  Wavelength (Å)  Resolution (Å)  Unit-cell parameters (Å, °)  Total reflections  Unique reflections  Average I/σ (I)  R_merge_ ^a^  Redundancy  Completeness (%)^b^ | PAL 5C beam line  P2_1_  1.00  30.00–1.90 (2.00–1.90)  *a* = 47.0, *b* = 48.3, *c* = 51.8, and β = 107.4  116286 (17553)  17080 (2483)  12.1 (7.0)  0.122 (0.294)  6.8 (7.1)  97.0 (97.1) | PAL 7A beam line  P2_1_  1.00  30.00–1.70 (1.73–1.70)  a = 47.0, b = 48.3, c = 51.8, and β = 107.4  171872 (7798)  23333 (1114)  53.8 (7.1)  0.053 (0.340)  7.4 (7.0)  98.2 (96.8) | PAL 7A beam line  P2_1_  1.00  49.53–1.68 (1.71–1.68)  a = 46.89, b = 47.60, c = 51.79, and β = 107.0  174733 (7172)  24925 (1221)  16.9 (5.8)  0.065 (0.193)  7.0 (5.9)  98.9 (96.8) | PAL 7A beam line  P2_1_  0.97934  30.00–1.70 (1.73–1.70)  *a* = 46.9, *b* = 47.9, *c* = 51.9, and β = 107.2  158545 (9014)  23880 (1252)  52.7 (20.3)  0.064 (0.192)  6.6 (7.2)  96.5 (99.8) |
| Refinement |  |  |  |  |
| Resolution range (Å)  No. of reflections of working set  No. of reflections of test set  No. of amino acid residues | 49.46–1.9 (1.95–1.9)  16230 (1184)  837(65)  210 | 48.43–1.69 (1.73–1.69)  22223 (1501)  1097(66)  211 | 49.53–1.68 (1.72–1.68)  23628 (1693)  1283(107)  211 | 49.57–1.69 (1.73–1.69)  22694 (1645)  1170 (79)  211 |
| No. of water molecules | 105 | 38 | 85 | 85 |
| R_cryst_ ^b^  R_free_ ^c^  R.m.s. bond length (Å)  R.m.s. bond angle (°)  Average B value (Å^2^) (protein)  Average B value (Å^2^) (solvent)  Ramachandran favored  Ramachandran outliers | 0.15 (0.164)  0.19 (0.21)  0.018  1.826  19.169  22.825  98.56  0 | 0.21 (0.32)  0.24 (0.31)  0.020  2.054  37.95  31.85  96.2  0 | 0.20 (0.26)  0.25 (0.29)  0.019  1.903  25.58  25.71  98.56  0 | 0.21 (0.24)  0.26 (0.30)  0.01  1.81  30.75  32.07  98.09  0 |

^a^ R_merge_ = ∑｜<I> - I｜/∑<I>.

^b^ R_cryst_ = ∑｜|Fo| - |Fc|｜/∑|Fo|.

^c^ R_free_ calculated with 5% of all reflections excluded from refinement stages using high-resolution data.

Values in parentheses refer to the highest resolution shells.
